# Substrate specificity of healthy human sera IgG antibodies with peroxidase and oxydoreductase activities

**DOI:** 10.1098/rsos.171097

**Published:** 2018-01-31

**Authors:** Anna S. Tolmacheva, Evgeny A. Ermakov, Valentina N. Buneva, Georgy A. Nevinsky

**Affiliations:** 1 Institute of Cytology and Genetics, Siberian Division of Russian Academy of Sciences, 10 Lavrentiev Avenue, Novosibirsk, Russia; 2 Siberian Division of Russian Academy of Sciences, Institute of Chemical Biology and Fundamental Medicine, 8 Lavrentiev Avenue, Novosibirsk 630090, Russia; 3 Novosibirsk State University, 2 Pirogova Street, Novosibirsk 630090, Russia

**Keywords:** abzymes, sera of healthy human, IgGs, oxidoreductase and peroxidase activities, substrate specificities

## Abstract

We have carried out an analysis of whether blood IgG antibodies can protect humans from oxidative stress by oxidizing different harmful compounds. A somewhat unexpected result was obtained. We show here for the first time that healthy human sera IgGs with the peroxidase (in the presence H_2_O_2_) efficiently oxidize different compounds: 3,3′-diaminobenzidine (**1**; DAB), 2,2′-azino-bis(3-ethylbenzothiazoline-6-sulfonic acid) diammonium salt (**2**; ATBS), *o*-phenylenediamine (**3**; OPD), homovanillic acid (**4**; HVA), α-naphthol (**5**), 5-aminosalicylic acid (**6**; 5-ASA) and 3-amino-9-ethylcarbazole (**7**; AEC), but seven of nine IgG preparations from different volunteers cannot oxidize *p*-hydroquinone (**8**: pHQ). The average apparent *k*_cat_ values in the H_2_O_2_-dependent oxidation by human IgGs decreased in the following order (min^−1^): ATBS (73.7) ≥ DAB (66.3) > AEC (38.0) ≥ HVA (19.8) ≥ α-naphthol (8.6) > OPD (0.62) ≥ 5-ASA (0.48) > pHQ (0.24). In the absence of H_2_O_2_ (oxidoreductase activity), the relative average *k*_cat_ values decreased in the following order (min^−1^): DAB (52.1) ≥ ATBS (50.5) > OPD (0.25). The peroxidase average activity of human IgGs was higher than the oxidoreductase one: 1.2-, 1.5- and 2.5-fold for DAB, ATBS and OPD, respectively. It should be assumed that antibodies can oxidize in addition to the large number of other different compounds analysed by us. As a whole, the specific wide repertoire of polyclonal human IgGs oxidizing various compounds could play an important role in protecting humans from oxidative stress and serve as an additional natural system destroying H_2_O_2_ and different toxic mutagenic and carcinogenic compounds.

## 1. Introduction

Partially reduced oxygen species, including ^•^OH, ^•^O_2_^–^ and H_2_O_2_, are known as potent oxidants of cellular proteins, DNA, RNA and lipids, and the most dangerous molecules of oxidative stress. Oxidative damage to cell components is ongoing and has been regarded as a significant factor in ageing due to mutagenesis and carcinogenesis [1–4]. In human and other organisms, the critical defence mechanisms for preventing oxidative stress are realized by several antioxidant enzymes: catalases, peroxidases, glutathione peroxidases and superoxide dismutases [5–8]. In the last two decades, it has been shown that IgG, IgA and IgM antibodies can possess a variety of different catalytic functions. Therefore, there is a very interesting question of whether immunoglobulins can have protective oxidoreductive functions. The abbreviations used in this article are given in table 1.

**Table 1.** Abbreviations.

Abzs	abzymes or catalytically active antibodies
Abs	antibodies
AI	autoimmune
ABTS	2,2^′^-azino-bis(3-ethylbenzothiazoline-6-sulfonic acid) diammonium salt
adrenaline	DL-epinephrine
AEC	3-amino-9-ethylcarbazole
5-ASA	5-aminosalicylic acid
DAB	3,3^′^-diaminobenzidine
HVA	homovanillic acid or 4-Hydroxy-3-methoxyphenylacetic acid
HSCs	hematopoietic stem cells
FPLC	fast protein liquid chromatography
OPD	*o*-phenylenediamine
pHQ	*p*-hydroquinone
αNpth	α-naphthol
HRP	horseradish peroxidase
SDS-PAGE	SDS - polyacrylamide gel electrophoresis

Artificial catalytic antibodies or abzymes (Abzs) against chemically stable analogues of transition states of chemical reactions catalysing more than 200 different reactions were well described [9–11]. Currently, natural IgGs and IgAs, sIgAs, and IgMs hydrolysing peptides, proteins, DNA, RNA or polysaccharides were found in the serum of patients with several autoimmune (AI) diseases (reviewed in [12–15]). There are only some exceptions of abzymes of healthy volunteers hydrolysing proteins and peptides [16,17] as well as polysaccharides [18] with very low activity. RNase, DNase, ATPase and protease activities of IgGs and/or IgMs of healthy humans are most often absent or extremely low, on the borderline of the sensitivity of the methods used [12–15]. However, there are examples of germline abzymes of healthy donors with catalytic activity. Germline Abs can exhibit high-level promiscuous amyloid- and superantigen-hydrolysing and/or autoantigen- and microbe-directed specificities [19,20]. In addition, abzymes of healthy volunteers with several oxidation–reduction activities are also described (see below). One cannot exclude that catalytic Abs in the healthy humans simply reflect constitutive production of germline antibodies as proved by the work of the Paul group [19,20].

It was shown that in the case of different AI experimental mice and human AI patient's abzymes with DNase, protease and amylase activities are the earliest and statistically significant markers of AI pathology onset and following development [12–15,21].

Natural Abzs of AI patients hydrolysing DNA, RNA, polysaccharides, oligopeptides and proteins are most often dangerous [12–15]. For example, multiple myeloma patients' Bence-Jones proteins [22], DNase abzymes from SLE [23] and MS [24] patients are cytotoxic, cause fragmentation of nuclear DNA and induce cell apoptosis stimulating acceleration of the development of these diseases. Myelin basic protein-hydrolysing Abs of MS and SLE patients can attack MBP of the myelin-lipoprotein sheath of axons and play a harmful role in the pathogenesis of these diseases [25–29]. Anti-VIP Abzs can have poor effect on pathogenesis due to a reduction in the concentration of VIP playing an important role in the asthma pathophysiology [16]. Increase in DNase activity of Abzs from Hashimoto thyroiditis patients correlate with decrease in the concentration of thyroid hormones and worsening of other immunological and biochemical indices [30]. The very widely used therapy of patients with thyroxine led only to a temporary change of the hormone concentration in the blood but did not affect the level of DNA-hydrolysing antibodies. However, the treatment of patients with an immunosuppressive drug plaquenil (7-chloro-4(beta-diethylamine-alpha-methylbutylamie) quinoline) leads to the decrease of DNase activity of Abzs with parallel increase in concentration of the thyroid hormones and progressive improvement in the clinical status of patients including reducing violations of the thyroid gland [30]. However, some protease Abs have a positive role. DNase IgGs from patients with several bacterial infections are not cytotoxic and can play a positive role in the primary line of protection against infections [13]. The increase in the relative activity of protease IgGs hydrolysing small peptides correlates with survival after sepsis [31]. IgGs of HIV-infected patients hydrolysing viral integrase and reverse transcriptase deprive these proteins catalytic activity and inhibit the development of AIDS [32,33].

Using experimental mice it was shown that during development of AI diseases, generation of pathogenic abzymes with different activities associated with significant changes in the profile of differentiation of bone marrow haematopoietic stem cells (HSCs) with parallel increase in proliferation of lymphocytes in different mouse organs [34,35]. Such changes are absent in healthy people and experimental mice, and therefore, if they have any abzymes, they in contrast to AI patients and mice are usually not pathogenic [12–15].

The first example of rabbit Abzs with superoxide dismutase activity was revealed in 1988 [36]. However, it was suggested that this activity might be due to traces of canonical enzymes with this activity. The existence of this activity was confirmed later; polyclonal and monoclonal Abs from various sources were shown efficiently to reduce singlet oxygen ${(}^{1}{{\text{O}}_{2}}^{*})$ to ^•^O_2_^–^ leading to the first intermediate H_2_O_3_ in a cascade of reaction finally resulting in H_2_O_2_ [37,38]. The superoxide dismutase activity of IgGs is linked with Fc, but not with their Fab-fragments [37,38]. However, because it is still the activity of antibodies, they can also be assigned to specific type of abzymes. These results suggest a possible protective function of these Abs and raise the question of whether a need in detoxification of ${(}^{1}{{\text{O}}_{2}}^{*})$ can play a critical role in the evolution of the immunoglobulin fold. These Abzs show a mechanism through which oxygen can be reduced and recycled in phagocyte action, thereby enhancing the microbicidal action of the immune system [37,38]. Even more surprising was the discovery of higher eukaryotes abzymes catalysing the formation of ozone used by cells during phagocytosis [39].

Superoxide dismutase, catalase, _22_-independent oxidoreductase and _22_-dependent peroxidase activities of polyclonal IgGs of healthy Wistar rats were analysed [39–44]. About 83% of Abs demonstrated superoxide dismutase activity, while only 17% of preparations possess catalase activity [44], but all IgGs oxidized 3,3′-diaminobenzidine (DAB) in the presence and the absence of hydrogen peroxide [40–45]. _22_-independent oxidoreductase and _22_-dependent peroxidase activities were higher in the presence of different metal ions [44].

IgGs from patients with viral hepatitis B or C and healthy donors were shown to possess comparable peroxidase activities [46,47]. Later more detail analysis of healthy human IgGs was performed [48]. After dialysis of IgGs against EDTA, the relative peroxidase activity dependently of individual IgGs decreased from 100 to approximately 10–85%, while oxidoreductase activity from 100 to 14–83%. Separation of Abs on Chelex non-charged and charged with Cu^2+^ ions results in separation of IgGs to many different subfractions having various levels of the specific oxidoreductase and peroxidase activities [48]. Among different metal ions, external Cu^2+^ ions were the best activators of these abzymes.

All IgGs of healthy Wistar rats oxidized not only DAB, but also other substrates with different efficiency: phenol, *o-*phenylenediamine (OPD), *p*-dihydroquinone, NADH, α-naphthol, but cannot oxidize adrenaline [42].

In the present report, we characterize for the first time the substrate specificity of the human abzymes with peroxidase and oxidase activities.

## 2. Results

### 2.1. Purification and characterization of IgGs

**Figure 1.**
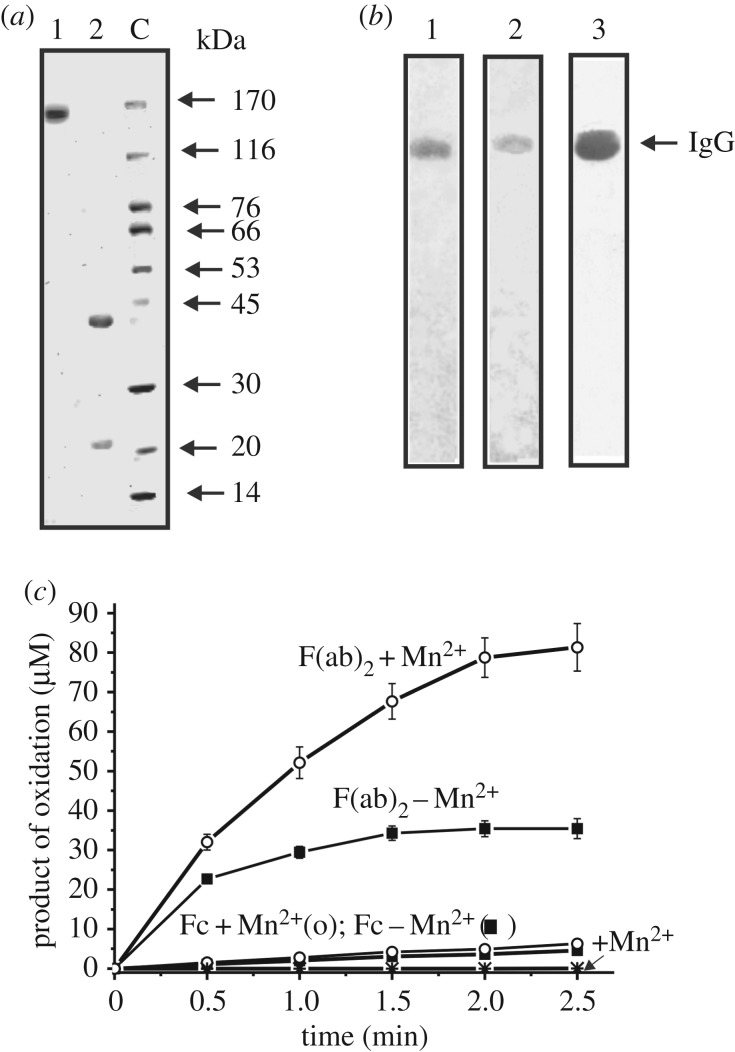
Analysis of IgG_mix_ (10 µg; an equimolar mixture of nine individual Abs) homogeneity by SDS-PAGE in 4–15% gradient gel (lane 1); IgG_mix_ before (lane 1) and after its boiling with DTT (lane 2) followed by silver staining of Abs (*a*). Lane C corresponds to protein molecular mass markers. *In situ* SDS-PAGE analysis of IgG_mix_ peroxidase (lane 1; 10 µg of IgGs) and oxidoreductase (lane 2; 15 µg of IgGs) activities by a non-reducing 4–15% gradient gel in the presence (lane 1) and in the absence of (lane 2) of H_2_O_2_ (*b*). After SDS-PAGE, the gels were incubated under special conditions for protein refolding. Then the oxidizing activities of IgG_mix_ were revealed by incubating longitudinal gel slices in the reaction mixture containing DAB and H_2_O_2_ (lane 1) or without hydrogen peroxide (lane 2). The control gels longitudinal slices were stained with Coomassie R250 (lane 3) to reveal the position of IgG_mix_. Relative activity of F(ab)_2_ and Fc fragments (0.2 mg ml^−1^) of IgG_mix_ in the oxidation of 0.93 mM DAB as well as spontaneous substrate oxidation in the absence of the fragments (*c*).

We have obtained electrophoretically homogeneous individual IgGs from sera of nine healthy volunteers as in [48]. All nine IgGs were electrophoretically homogeneous. Figure 1 demonstrates SDS-PAGE analysis of an equimolar mixture of nine IgGs (IgG_mix_). One can see that IgG_mix_ demonstrates a single band of the typical 150 kDa IgG before (lane 1) and two bands corresponding to the L and H and chains (lane 2) after Abs reduction with DTT (silver staining; figure 1*a*). It was previously shown that all IgG preparations purified by affinity chromatography on Protein G-Sepharose with following FPLC gel filtration oxidize DAB and do not contain any canonical enzymes [48]. This new set of nine IgGs was used for analysis of substrate specificity in the oxidation of several different typical substrates of various H_2_O_2_-dependent peroxidases and H_2_O_2_-independent oxidoreductases (designated as peroxidases and oxidoreductases, respectively).

To prove that oxidoreductase and peroxidase activities of IgG_mix_ are its intrinsic properties several strict criteria were validated similar to [48]. IgG_mix_ was electrophoretically homogeneous (figure 1*a*). It was shown that similar to [48] after FPLC gel filtration of IgG_mix_ in the acidic buffer (pH 2.6), the peaks of peroxidase and oxidoreductase activities tracked exactly with intact IgG_mix_. Sepharose bearing immobilized mouse Abs against human IgGs completely absorbed both activities and their peaks coincided with IgG_mix_ eluted by acidic buffer (data not shown). To exclude possible traces of contaminating canonical peroxidases and oxidoreductases, the IgG_mix_ was subjected to *in situ* SDS-PAGE, and its peroxidase and oxidoreductase activities were detected by the gel incubation using the standard mixture containing DAB. Yellow-brown bands were detected only in the position of intact IgG_mix_ (figure 1*b*). As SDS usually dissociates all complexes of protein, the revealing of the peroxidase and oxidoreductase activities in the gel fragments corresponding only to the IgG_mix_ and the absence of any other bands of the activities or proteins, provides direct evidence that IgG_mix_ possesses these activities.

It is noted above that the superoxide dismutase activity is exhibited by the Fc fragments of antibodies [37,38]. We compared the relative peroxidase and oxidoreductase activities of F(ab)_2_ and Fc of IgG_mix_. It was shown that F(ab)_2_ fragments possess the peroxidase and oxidoreductase activities approximately 21- to 22-fold higher than Fc fragments in the presence or absence of metal ions (e.g. figure 1*c*). Consequently, these activities are mainly determined by the variable regions of the abzymes.

### 2.2. Substrate specificities of human IgGs

**Figure 2.**
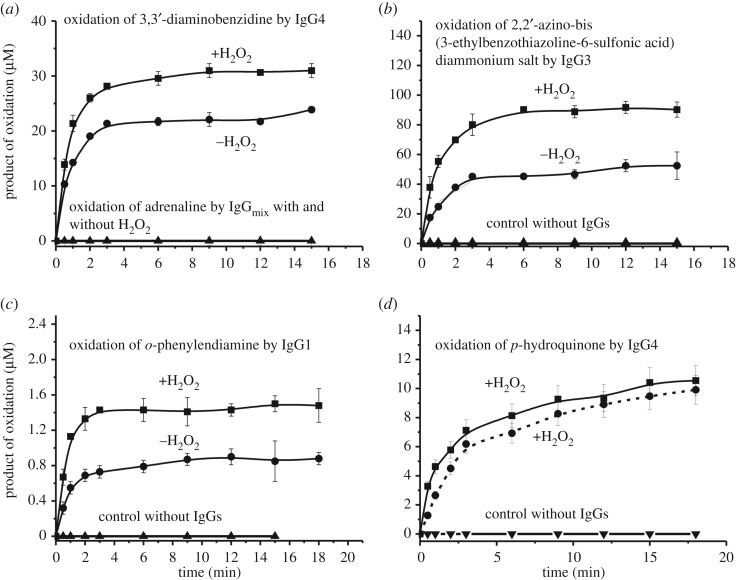
Typical examples of the time-dependences of products accumulation of oxidation by various IgGs of 3,3′-diaminobenzidine (0.93 mM; 0.2 mg ml^−1^ IgGs) and adrenaline (0.15 mM, 0.5 mg ml^−1^ IgGs), 2,2′-azino-bis(3-ethylbenzothiazoline-6-sulfonic acid) diammonium salt (0.36 mM; 0.2 mg ml^−1^ IgGs), *o-*phenylenediamine (0.19 mM; 0.5 mg ml^−1^ IgGs) and *p*-hydroquinone (0.12 mM; 0.5 mg ml^−1^ IgGs), in the presence and in the absence of H_2_O_2_.

**Figure 3.**
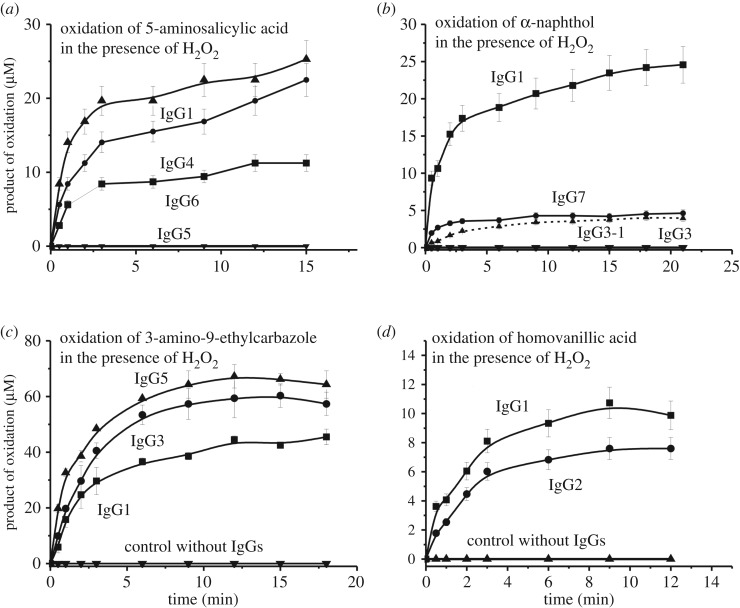
Typical examples of the time-dependences of products accumulation in the reaction of four substrates oxidation by various individual IgGs in the presence of H_2_O_2_: 5-aminosalicylic acid (0.38 mM; 0.5 mg ml^−1^ IgGs), α-naphthol (0.07 mM; 0.1 mg ml^−1^ IgGs), 3-amino-9-ethylcarbazole (0.19 mM; 0.1 mg ml^−1^ IgGs) and homovanillic acid (0.55 mM; 1.0 mg ml^−1^ IgGs).

IgGs of different donors oxidized several compounds in the presence and/or in the absence of H_2_O_2_. The affinity of all potential substrates to antibodies was relatively low, and the achievement of their concentrations close to saturation in most cases was impossible due to the low solubility of the compounds. Taking this into account, the kinetic curves were obtained using for each substrate feasible maximal concentrations giving no precipitates during the reaction. To estimate the activities quantitatively, we have found the concentration for each IgG preparation corresponding to the pseudo-first order of the reaction conditions within the linear regions of the time courses and Ab concentration curves. Figure 2 demonstrates typical time-dependences of oxidized product accumulation of four substrates in the reactions catalysed by various individual IgGs in the presence and in the absence of H_2_O_2_. Figure 3 shows time-dependences of four other substrates oxidation by various individual IgGs in the presence of hydrogen peroxide. Such dependencies were obtained for all nine substrates used in the case of all nine IgG preparations. The efficiency of the Ab-dependent oxidation of various substrates in the presence and the absence of hydrogen peroxide very much depended on both the substrate type and antibody preparations used including their concentrations (figures 2 and 3). It was previously shown that IgG preparations from sera of Wistar rats are inactive in the oxidation of adrenaline [42]. Human IgG_mix_ was also inactive in the H_2_O_2_-dependent and independent oxidation of adrenaline (figure 2*a*)_._

The relative apparent *k*_cat_ values (*k*_cat_ = *V* (M min^−1^)/[IgG] (M)) characterizing oxidation of eight substrates in their fixed concentrations were determined. The data are summarized in tables 2 and 3. The ratio of peroxidase and oxidoreductase activities for nine individual IgGs and eight substrates was very different. In the absence of hydrogen peroxide, all nine preparations were inactive in the oxidation of homovanillic acid (HVA, table 2), α-naphthol, 5-aminosalicylic acid (5-ASA) and 3-amino-9-ethylcarbazole (AEC) (table 3). In addition, only two of nine IgGs oxidized *p*-hydroquinone (pHQ) in the presence and in the absence of H_2_O_2_ (table 3).

**Table 2.** The apparent *k*_cat_ values characterizing peroxidase and oxydoreductase activity of individual IgGs from the sera of healthy donors in the oxidation of DAB, ATBS, OPD and HVA.

	*k*_cat_, min^−1^^a^							
	DAB		ABTS		OPD		HVA	
	+H_2_O_2_	−H_2_O_2_	+H_2_O_2_	−H_2_O_2_	+H_2_O_2_	−H_2_O_2_	+H_2_O_2_	−H_2_O_2_
IgG number	1	2	3	4	5	6	7	8
IgG-1	54.2^b^	49.0	93.7^b^	57.9	0.64	0.24	22.2	∼0.0^c^
IgG-2	69.4	41.8	81.6	60.5	0.79	0.21	11.5	∼0.0
IgG-3	70.5	55.4	76.7	36.6	0.68	0.15	27.9	∼0.0
IgG-4	51.6	54.7	59.9	34.9	0.73	0.21	26.4	∼0.0
IgG 5	92.8	58.0	54.7	34.9	0.22	0.19	13.2	∼0.0
IgG-6	60.5	56.6	72.3	61.3	0.36	0.17	21.0	∼0.0
IgG-7	65.3	49.4	81.5	69.5	0.61	0.28	16.2	∼0.0
IgG-8	69.3	48.1	66.0	48.9	0.82	0.29	17.9	∼0.0
IgG-9	63.3	56.1	76.5	46.9	0.71	0.55	21.6	∼0.0
average value	66.3 ± 12.0	52.1 ± 5.3	73.7 ± 12.0	50.5 ± 12.9	0.62 ± 0.20	0.25 ± 0.12	19.8 ± 5.6	∼0.0
corr. coeff.^d^	1–2 (parameter numbers) = +0.18; 1–3 = −0.47; 1–5 = −0.51; 1–7 = −0.58; 2–4 = − 0.58; 2–6 = +0.05;							
	3–4 = +0.67; 3–5 = +0.36; 3–7 = +0.06; 4–6 = +0.08; 5–6 = +0.32; 5–7 = +0.22							
differences, *p*^e^	1–3 = 0.13; 2–4 = 0.86; *p* corresponding to other sets of parameters of this table 2, <0.05 (0.0004–0.0081)							

^a^The apparent *k*_cat_ values of the reaction at fixed concentration of H_2_O_2_ (10 mM) and different concentration of substrates: DAB (0.93 mM), ATBS (0.36 mM), HVA (0.55 mM) and α-naphthol (0.07 mM) were calculated using average relative activity (RA) values: *k*_cat_* = V* (M min^−1^)/ [IgGs] (M).

^b^For each value, a mean of three measurements is reported; the error of the determination of values did not exceed 7–15%.

^c^In the absence of H_2_O_2_ there was not observed visible oxidation of HVA.

^d^The CCs between the sets of parameters denoted by numbers 1–7 are given.

^e^The difference (*p*) was estimated Mann–Whitney test, *p* < 0.05 was considered statistically significant.

**Table 3.** The apparent *k*_cat_ values characterizing peroxidase and oxydoreductase activity of individual IgGs from the sera of healthy donors in the oxidation of α-naphthol, 5-ASA, AEC and pHQ.

	apparent *k*_cat_, min^−1^ at fixed concentrations of substrates^a^^,^^b^							
	α-naphthol		5-ASA		AEC		pHQ	
	+H_2_O_2_	−H_2_O_2_	+H_2_O_2_	−H_2_O_2_	+H_2_O_2_	−H_2_O_2_	+H_2_O_2_	−H_2_O_2_
IgG number	9	10	11	12	13	14	15	16
IgG-1	18.9	∼0.0	0.16	∼0.0^c^	26.0	∼0.0	∼0.0	∼0.0
IgG-2	5.3	∼0.0	0.0	∼0.0	32.6	∼0.0	∼0.0	∼0.0
IgG-3	0.0	∼0.0	0.93	∼0.0	48.9	∼0.0	∼0.0	∼0.0
IgG-4	7.6	∼0.0	0.18	∼0.0	43.1	∼0.0	∼0.0	∼0.0
IgG 5	3.4	∼0.0	0.0	∼0.0	25.2	∼0.0	∼0.0	∼0.0
IgG-6	26.9	∼0.0	0.45	∼0.0	45.7	∼0.0	∼0.0	∼0.0
IgG-7	7.9	∼0.0	0.35	∼0.0	44.3	∼0.0	1.12	2.30
IgG-8	5.3	∼0.0	2.03	∼0.0	36.9	∼0.0	0.0	∼0.0
IgG-9	1.7	∼0.0	0.19	∼0.0	39.6	∼0.0	0.77	0.92
average value	8.6 ± 8.5	∼0.0	0.48 ± 0.65	∼0.0	38.0 ± 8.5	∼0.0	0.24 ± 0.42	0.36 ± 0.78
CCs table 3	9–11 (parameter numbers) = −0.14; 9–13 = −0.015; 11–13 = +0.29							
CCs tables 2 and 3^d^	1–9 = −0.46 (parameter numbers); 1–11 = +0.03; 1–13 = −0.37; 3–9 = +0.29; 3–11 = −0.15;							
	3–13 = −0.03; 5–9 = −0.35; 5–11 = +0.38; 5–13 = +0.22; 7–9 = +0.07; 7–11 = +0.17; 7–13 = +0.56							
differences, *p*^e^	8–10 = 1.0; 5–11 = 0.93; 8–12 = 1.0; 10–12 = 1.0; 8–14 = 1.0; 10–14 = 1.0; 12–14 = 1.0; 16–6, 8, 10, 12, 14, 15							
	= 0.052–0.96. The *p*-values corresponding to other sets of parameters of tables 2 and 3 were < 0.05							
	(0.0004–0.013)							

^a^For each value, a mean of three measurements is reported; the error of the determination of values did not exceed 7–15%.

^b^The apparent *k*_cat_ values of the reaction at fixed concentration of H_2_O_2_ (10 mM) and different concentration of substrates: OPD (0.02 mg ml^−1^ or 0.185 mM), 5-ASA (0.38 mM), AEC (0.19 mM) and pHQ (0.124 mM) were calculated using average RA values: *k*_cat_* = V* (M min^−1^)/[IgGs] (M).

^c^In the absence of H_2_O_2_ there was not observed visible oxidation of α-naphthol, 5-ASA and AEC.

^d^The CCs between the sets of parameters denoted by numbers 1–7 in table 2 and numbers 9, 11, 13 in table 3 are given.

^e^The difference (*p*) was estimated Mann–Whitney test, *p* < 0.05 was considered statistically significant.

The correlation analysis of the data on the relative activity of antibodies in the hydrolysis of different substrates (the sets of these *k*_cat_ are indicated in tables 2 and 3 with numbers from 1 to 16) was carried out. Interestingly, several IgGs demonstrated negative correlation in the oxidation of different substrates in the presence of hydrogen peroxide: DAB-ATBS (−0.47), DAB-OPD (−0.51), DAB-HVA (−0.58) (table 2), DAB-α-naphthol (−0.46), DAB-AEC (−0.37) (tables 2 and 3), α-naphthol-5-ASA (−0.14) and α-naphthol-AEC (−0.015) (table 3). However, several preparations display positive but weak correlation: 5-ASA with DAB (+0.03), AEC (+0.29), OPD (+0.38), HVA (+0.17), as well as for ATBS-α-naphthol (+0.29), OPD-AEC (+0.22). The maximal positive correlation was observed for HVA-AEC (+0.56), while negative one for DAB-HVA (−0.58) (tables 2 and 3).

The relative average apparent *k*_cat_ values in the presence of hydrogen peroxide decreased in the following order (min^−1^): ATBS (73.7) ≥ DAB (66.3) > AEC (38.0) ≥ HVA (19.8) ≥ α-naphthol (8.6) > OPD (0.62) ≥ 5-ASA (0.48) > pHQ (0.24) (tables 2 and 3). In the absence of H_2_O_2_, the relative average apparent *k*_cat_ values decreased in the following order (min^−1^): DAB (52.1) ≥ ATBS (50.5) > OPD (0.25), while five other compounds were not substrates for IgGs with oxidoreductase activity (tables 2 and 3). The average peroxidase activity of IgGs was higher than the oxidoreductase one: 1.2-, 1.5- and 2.5-fold for DAB, ATBS and OPD, respectively (table 2).

The relative activities for sets of individual IgGs in the hydrolysis of different substrates do not correspond to normal Gaussian distribution. Therefore, the difference between various sets was estimated using the Mann–Whitney test. For most sets of relative activities corresponding to various substrates in the presence and absence of hydrogen peroxide (totally 66 *p-*values), there were statistically significant differences (51 values; *p* = 0.0004–0.013), while 15 *p*-values were greater than 0.05 (0.052–1.0; tables 2 and 3).

**Figure 4.**
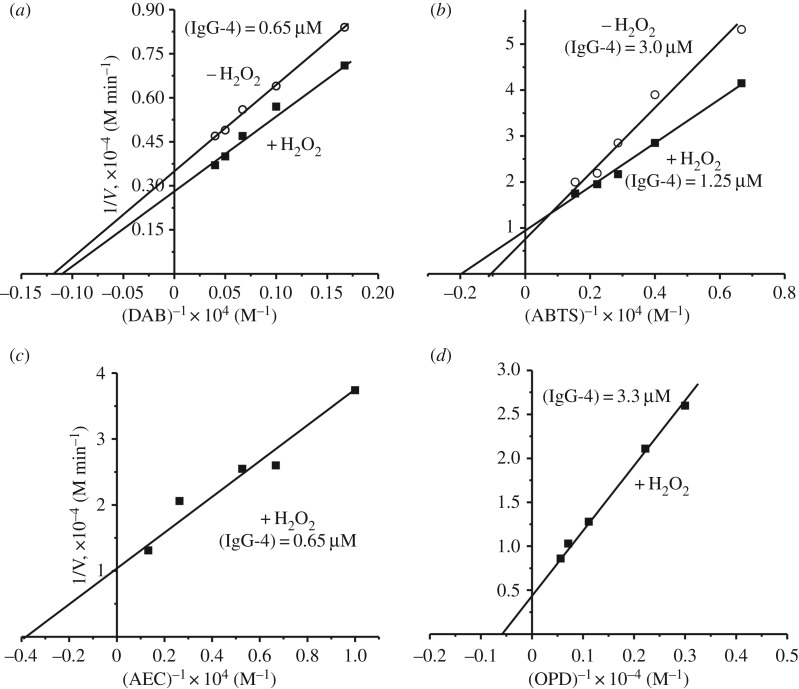
Evaluation of the *K*_m_ and *V*_max_ values for hydrolysis of several substrates by IgG-4 using Lineweaver–Burk plots in the presence and/or in the absence of H_2_O_2_: DAB (*a*), ATBS (*b*), AEC (*c*) and OPD (*d*).

For a more precise definition of a possible affinity of various compounds for abzymes the *K*_m_ and *V*_max_ (*k*_cat_) values characterizing Ab-dependent oxidation of good substrates were measured in the case of one preparation, IgG-4 (figure 4). All data obtained are summarized in table 4. Interestingly, the *K*_m_ values for DAB, ATBS, AEC and OPD are to some extent comparable, as well as the apparent *k*_cat_ values for DAB and ATBS in the presence and the absence of H_2_O_2_ (table 4). However, the *k*_cat_ values for AEC and OPD are significantly different. At the same time, *k*_cat_ values determined from dependencies of 1/*V* upon 1/[Substrate] (table 4) are in good agreement with the apparent *k*_cat_ values estimated using fixed concentration of these substrates (table 2).

**Table 4.** The *K*_m_ and *k*_cat_ values characterizing oxidation of four different substrates by IgG-4*

Substrate	Conditions	*K*_m_, M	*k*_cat_, min^−1^
DAB	+H_2_O_2_	(9.3 ± 0.8) × 10^−4^	57.0 ± 5.0**
	−H_2_O_2_	(8.5 ± 0.7) × 10^−4^	44.0 ± 4.0
ATBS	+H_2_O_2_	(5.3 ± 0.4) × 10^−4^	64.0 ± 6.0
	−H_2_O_2_	(8.9 ± 0.6) × 10^−4^	41.6 ± 3.5
AEC	+H_2_O_2_	(2.7 ± 0.3) × 10^−4^	153 ± 15.0
OPD	+H_2_O_2_	(1.8 ± 0.15) × 10^−3^	0.78 ± 0.06

*For each value, a mean of three measurements is reported; the error of the determination of values did not exceed 7–15%.

**The apparent *k*_cat_ values of the reaction in the absence (−H_2_O_2_) or at fixed concentration of +H_2_O_2_ (10 mM) were calculated: *k*_cat_ = *V* (M min^−1^)/[IgGs] (M).

## 3. Discussion

Artificial Abzs usually demonstrated 10^2^- to 10^6^-fold lower *k*_cat_ values than those for canonical enzymes [12–15]. The apparent *k*_cat_ values for natural abzymes from sera of AI patients varied with some exception in the range 10^−4^–40 min^−1^ [12–15]. Thus, the average peroxidase (66.3 ± 12 min^−1^) and oxidoreductase (52.1 ± 5.3 min^−1^) activities of healthy humans polyclonal IgGs in the DAB oxidation are significantly higher than those of most known natural Abzs. Most of the canonical peroxidases including the horseradish one (HRP) can oxidize many very different compounds in the absence and the presence of H_2_O_2_ [6–8]. The *k*_cat_ value in HRP-dependent DAB peroxidase oxidation was estimated earlier as 1.1 × 10^4^ min^–1^; the *k*_cat_ of its oxidoreductase activity was 24-fold lower (4.5 × 10^2^ min^–1^) [42].

Interestingly, the *k*_cat_ of IgGs from sera of 11 Wistar rats in DAB oxidation in the presence of hydrogen peroxide varied from 1.8 × 10^2^ to 2.9 × 10^3^ min^−1^ (average value 9.6 × 10^2^ min^−1^), while in the absence of H_2_O_2_ from 91 to 3.6 × 10^3^ min^−1^ (7.2 × 10^2^ min^−1^) [41]. Thus, the average peroxidase activity of Wistar rat antibodies is approximately 11.4-fold lower than that of HRP, while oxidoreductase activity is 1.6-fold higher; for several individual Wistar rats, it is 3.8–8.0 times greater than that for HRP [41]. HRP oxidizes DAB with and without H_2_O_2_ approximately 173- and 8.6-fold, respectively, faster than polyclonal human IgGs (according to the average values for IgGs, table 2). Interestingly, polyclonal rat IgGs demonstrate 14.5-fold higher average *k*_cat_ value in DAB oxidation in the presence and 13.8-fold greater activity in the absence of hydrogen peroxide compared to those for human IgGs. The *k*_cat_ values for polyclonal human and Wistar rat IgGs were calculated using their total concentrations. Since the specific activities were calculated using the total concentration of polyclonal IgGs and only a small fraction of IgGs possesses peroxidase- or oxidase-like activities [40–43], the specific activities of monoclonal IgGs possessing these activities may be significantly higher compared with total IgGs analysed by us (tables 2–4).

As shown earlier, abzymes with several enzymatic activities are the earliest indicators of development of different AI diseases, while some of them are absent in the blood of healthy donors [11–15]. It was shown that DNase and RNase abzymes of AI patients present a ‘cocktail’ of Abs directly to DNA and RNA and anti-idiotypic Abs against active centres of DNase I, DNase II, RNase and other enzymes hydrolysing nucleic acids [11–15]. Abzymes of AI patients with proteolytic and oligosaccharide-hydrolysing activities are Abs against different proteins and oligosaccharides [11–15]. For now it is not clear what antigens in healthy donors can stimulate the formation of abzymes with oxidoreductase and peroxidase activities.

An ever-increasing number of investigations suggests that AI diseases originate from defects in the HSCs [49]. It is known that apoptotic cells are the primary source of antigens and immunogens in SLE and other AI diseases, which trigger the recognition, perception, processing and/or presentation of apoptotic autoantigens by antigen-presenting cells, and can cause AI processes [50]. In addition, the appearance of abzymes in SLE and encephalomyelitis AI mice is associated with significant changes in profile of differentiation and level of proliferation of mice bone marrow HSCs as well as with increase in the level of lymphocyte proliferation in different organs [21,34,35]. However, production of abzymes may also be not associated with defects of the HSCs. Immunization of healthy non-AI mice with DNA or other antigens also leads to the production of catalytic antibodies [21,34]. But this process is not associated with the changes in the profile of differentiation of mice bone marrow HSCs; it is the result of further differentiation of previously differentiated cells in bone marrow and suppression of the level of apoptosis of lymphocytes in different organs [21,34]. Most probably, in healthy humans the production of abzymes with peroxidase and oxidoreductase activities is not associated with any changes in the profile of differentiation of bone marrow HSCs. This process may be stimulated by different toxic, mutagenic and carcinogenic compounds falling into human organisms and stimulating lymphocytes' additional differentiation and increase in their proliferation in different organs. In addition, anti-idiotypic Abs against active centres of various enzymes can also possess different catalytic activities [11–15]. Thus, one cannot exclude a possibility of the formation of different catalase and oxidoreductase abzymes may be due to production of anti-idiotypic Abs against canonical catalase, glutathione peroxidase, superoxide dismutase and other enzymes oxidizing different substrates in parallel with formation of Abs to different dangerous compounds. Tables 3 and 4 demonstrate the catalytic diversity of abzymes towards various substrates in the case of different humans. One cannot exclude that it may be a consequence of the fact that various harmful substances enter the organisms of different people, which can lead to the production of abzymes with a different substrate specificity. As was mentioned above, HRP can oxidize different compounds mostly in the presence of H_2_O_2_ and to a much lesser extent in its absence. At this time, it is not clear whether the same or different molecules of human IgGs oxidize substrates in the presence and in the absence of hydrogen peroxide. However, the same IgG preparations oxidize three substrates in the presence and absence of hydrogen peroxide, while another five only in the presence of H_2_O_2_ (tables 2 and 3). Therefore, one can propose that in the case of the same IgG preparations substrate specificity of peroxidase H_2_O_2_-dependent activity may be advanced comparing with their oxidoreductase activity.

Here we demonstrate for the first time that the peroxidase and oxidoreductase activities of human IgGs can effectively oxidize not only DAB but also other typical substrates of various enzymes with oxidative activities. Sera of humans contain Abzs with superoxide dismutase activity. Therefore, Abzs with superoxide dismutase activity can reduce oxygen from ^•^O_2_^–^ to H_2_O_2_, while peroxidase Abs can neutralize hydrogen peroxide and in parallel destroy harmful compounds. Our data indicate that substrate specificity of human IgGs peroxidase may be more expanded than its oxidoreductase specificity. Taken together, we suggest that the specific repertoire of polyclonal human Abs can serve as an additional natural system of reactive oxygen species detoxification and Abs can destroy hydrogen peroxide, mutagenic, toxic and carcinogenic compounds.

## 4. Material and methods

### 4.1. Chemicals, donors and patients

The chemicals including substrates used in this work were mainly from Sigma or Sigma-Aldrich: HVA, 4-hydroxy-3-methoxyphenylacetic acid (Sigma, H1252), *o*-phenylenediamine (Sigma, P9029), hydrogen peroxide (Sigma, H1009), ammonium persulfate (Sigma, A3678), pepsin (Sigma, 77151), DAB (Sigma -Aldrich, D8001), α-naphthol (Sigma-Aldrich, N1000) and *ρ*-hydroquinone (Sigma-Aldrich, H17902).

Several chemicals were from other manufacturers: 5-ASA (Acros organics, 134330050), AEC (Calbiochem, 152224), dl-epinephrine (MP Biomedicals, 151064), Tris (MP Biomedicals, 11TRIS01KG), NaCl (MP Biomedicals, 0219484801), Coomassie Brilliant Blue R 250 (MP Biomedicals, 821616), K_2_HPO_4_ (Amresco, Am-0705), KH_2_PO_4_ (Amresco, Am-0781), Triton X-100 (Amresco, Am-0694) and acrylamide (AppliChem, A1089, 0500).

All sorbents were from GE Healthcare: HiTrap Protein G HP column (GE Healthcare, 17-0404-01), HiTrap Protein A HP column (GE Healthcare, 17-0402-01) and Superdex 200 HR column (GE Healthcare, 17-5175-01).

The sera of nine healthy humans (19–45 years old) were used to study Abzs. The healthy donors had no history of rheumatologic, respiratory, AI, gastrointestinal, reproductive, cardiovascular or nervous system pathologies.

Electrophoretically homogeneous IgGs were purified by sequential affinity chromatography of volunteer's serum proteins on protein G-Sepharose and following FPLC gel filtration on the column with Superdex 200 HR 10/30 in the acidic buffer [48]. SDS-PAGE analysis of IgG under non-reducing and reducing conditions was carried out using 4–15% gradient gels (0.1% SDS) according to Laemmli with following silver staining as in [48].

### 4.2. Assay of activities

Measurements of Ab's oxidoreductase and peroxidase activities were performed using optimal conditions. It should be mentioned that nine different potential substrates possess very different solubility and the efficiency of their oxidation by various antibodies. Therefore, the analysis of the specificity of different substrates was carried out using different concentrations of both substrates and abzymes. Reaction mixtures (100–150 µl) contained 25 mM of K-phosphate (pH 6.8), with or without 10 mM H_2_O_2_, one of different substrates (0.07–0.55 mM) and 0.05–0.5 mg ml^−1^ IgGs.

Oxidation of DAB, 5-ASA, 2,2′-azino-bis(3-ethylbenzothiazoline-6-sulfonic acid) diammonium salt (ABTS), adrenaline, AEC and *o*-phenylenediamine (OPD) were detected from the changes in optical density at 450 nM (A_450_) using 0.1 cm quartz cuvettes and Genesis 10S Bio spectrophotometer (Thermo Scientific, USA). Reaction mixtures were incubated at 22°C and time dependencies (0.5–20 min) of A_450_ change were analysed. Oxidation of α-naphthol (α-Npth), HVA and pHQ was analysed at 22°C by measuring the change in fluorescence (ΔF) in 5 mm thermostated quartz cuvettes using a Varian Cary Eclipse Fluorescence Spectrophotometer (Agilent Technologies, USA). Excitation was performed at 324 nm (α-Npth), 315 nm (HVA) or 300 nm (pHQ), and the fluorescence emission was detected at 458 nm, 425 nm or 330 nm, respectively.

### 4.3. *In situ* analysis of catalytic activities

SDS-PAGE analysis of IgG_mix_ (10 µg/protein; an equimolar mixture of nine preparations) activities under non-reducing conditions was carried out in 4–15% gradient gels (0.1% SDS) using the Laemmli system with silver staining [40–45]. To recover the enzymatic activity after Abs electrophoresis, SDS was removed by the gel incubation at 22°C for 1 h with K-phosphate (pH 6.8). The gel was treated five times for 5 min with this buffer. To assay for catalytic activities, the gel longitudinal slices were incubated using the standard reaction mixture containing 0.2 mg ml^−1^ DAB and 10 mM H_2_O_2_ (or without) for 15–44 h at 22°C. The parallel longitudinal gel lanes were used for detection of the IgG position by their Coomassie R250 staining. Yellow-brown bands of a coloured product of DAB oxidation were revealed only in the position of intact IgG_mix._

### 4.4. Preparation of F(ab)_2_ fragments

To obtain the F(ab)_2_ fragments, IgG_mix_ was used. IgG_mix_ was cleaved with pepsin as in [51] and its fragments were purified by affinity chromatography on protein A-Sepharose. The F(ab)_2_ fractions eluted upon application contained F(ab)_2_ and pepsin. They were concentrated and fractionated using gel filtration on a Superdex 200 HR 10/30 column equilibrated TBS buffer. The fraction of F(ab)_2_ fragments was collected and dialysed against 20 mM Tris–HCl (pH 7.5). The F(ab)_2_ fragment was electrophoretically homogeneous.

### 4.5. Kinetic analysis

The *K*_M_ and *V*_max_ (*k*_cat_) values were calculated from the dependencies of *V* versus [Substrate] by nonlinear least-squares fitting using Microcal Origin v. 5.0 software and presented as linear transformations using a Lineweaver–Burk plot [52]. The *K*_m_ and *k*_cat_ values are reported as the mean ± standard deviation of three independent experiments for each substrate and IgG preparation. Errors in the values were within 7–15%. Correlation coefficients (CCs) were estimated using Microsoft Excel test system. To check for normality of the values distribution the criterion of Shapiro–Wilk's *W* test was used. Most of the sample sets did not fit the normal Gaussian distribution; the differences between different groups of IgG samples and substrates were analysed by the Mann–Whitney test. The non-parametric ranking method of Spearman was used for the correlation analysis.

## Data accessibility

The data supporting the findings of this study are available on Figshare (https://doi.org/10.6084/m9.figshare.5446258.v1) [53].
